# Scaling of speed with group size in cooperative transport by the ant *Novomessor cockerelli*

**DOI:** 10.1371/journal.pone.0205400

**Published:** 2018-10-09

**Authors:** Aurélie Buffin, Takao Sasaki, Stephen C. Pratt

**Affiliations:** 1 Mesa Community College, Mesa, Arizona, United States of America; 2 Odum School of Ecology, University of Georgia, Athens, Georgia, United States of America; 3 School of Life Sciences, Arizona State University, Tempe, Arizona, United States of America; Universidade de São paulo, BRAZIL

## Abstract

Working together allows social animals to accomplish tasks beyond the abilities of solitary individuals, but the benefits of cooperation must be balanced with the costs of coordination. Many ant species form cooperative groups to transport items too large for a single ant. However, transport by groups is often slower and less efficient than that of lone ants, for reasons that remain poorly understood. We tested the hypothesis that groups are slower when porters must encircle the load to carry it, because this arrangement places ants in a variety of postures relative to the load and the direction of travel. Porters may therefore have difficulty maximizing individual forces and aligning them with those of other group members. Experiments on the desert ant *Novomessor cockerelli*, an adept cooperative transporter, did not support this hypothesis. Groups ranging in size from one to four ants were induced to carry loads such that all porters were aligned with one another. Load weight was adjusted so that all porters pulled the same per capita weight, but lone porters were nonetheless faster than groups of any size. As group size increased, porters persisted in carrying the load for longer periods before letting go. We used simulations to explore a scenario in which ants vary in their intrinsic speed and the group's speed is limited by that of its slowest member. This proposed mechanism is analogous to other social groups where group efficiency is determined by the weakest link. We discuss how interactions among porters, mediated by the load itself, might explain such a constraint.

## Introduction

Group living presents animals with both benefits and costs. Benefits include buffering of environmental variation [1,2], improved detection of predators and prey [3–6], and opportunities to cooperate on tasks beyond the capacity of a solitary individual [7,8]. Costs include competition among group members for limited resources and enhanced transmission of pathogens and parasites [9–11]. Both benefits and costs scale with group size in ways that can be complex and nonlinear, posing a challenge to the prediction of optimal size [12–14].

Group size effects are especially complex in eusocial insects such as ants and bees, whose highly coordinated behavior often depends on the number of participating workers [15]. Larger numbers enhance many features of social coordination, including the emergence of division of labor [16–18], the generation of complex social foraging tactics [19–21], the buffering of stochastic variation in food supplies [22–24], and the regulation of internal features such as brood temperature [25]. At the same time, larger worker populations can pose challenges to the optimal allocation and coordination of labor. If too many workers are assigned to one task, this may cause mutual interference among workers [26,27] as well as diverting workers from other tasks where they might be more efficiently employed [28].

In this study, we examined how group size affects the efficiency of cooperative food transport, a collective behavior that has evolved independently in many ant genera [29–31]. Cooperation allows colonies to retrieve food items too big for a single porter, with potential benefits that include broadening the colony's dietary range, evading competitors for rich items, and enhancing the efficiency with which food is moved. In some species transport is "superefficient," meaning that the per capita weight moved by porters increases with group size, without any loss of speed [32–34]. As a result, larger groups have a higher per capita rate of food delivery to the colony than do smaller groups or lone porters [35]. This advantage has been shown only for swarm raiders such as the army ants and driver ants [33], and it may be associated with their distinctive mode of transport: porters straddle the load and face forward, allowing them to run in a common direction and in their normal posture. The leading ant, typically the largest in the group, bears the bulk of the weight, while those behind her work to counter rotational forces and to prevent the load from dragging [36]. The resulting enhancement of efficiency may contribute to the success of these ants' distinctive foraging strategy, in which populous societies are fueled by copious arthropod prey delivered over large trail networks [33,37,38].

Outside of the swarm-raiding ants, cooperative transport allows foragers of many species to carry heavier loads than can a single ant, but at the cost of lower velocity. As a result, these ants are not superefficient: their per capita rate of prey delivery is lower for groups than for individuals [35,39–41]. At a functional level, this disadvantage is likely more than made up by the benefit of removing rich prey from competitors [39,41,42]. At a proximate level, however, the reasons for the decline in efficiency with group size remain unknown. A possible explanation concerns the posture of cooperative transporters in these species. Unlike the swarm raiders, most of them encircle their load rather than straddling it, placing them in a wide variety of stances relative to the direction of travel [29]. This arrangement may place some ants in less advantageous positions for exerting a force on the load if, for example, transport efficiency is reduced when walking sideways. Encircling the load may even lead ants to cancel each other's efforts if the forces they exert are out of alignment with one another [43–45]. As a result, the net force exerted by the group will be less than the sum of the members’ potential contributions, and groups will move more slowly than individuals, even when carrying the same per capita weight.

On the other hand, mere alignment of porters may not be enough to achieve higher efficiency. Superefficiency may also depend on the details of group member interactions, such as the distinct roles played by leading and following porters in army ants [36]. For example, if ants vary in transport ability, some group members may be forced to perform below their capacity, so that they remain synchronized with less capable teammates.

We studied the role of porter alignment in cooperative transport by *Novomessor cockerelli*. These desert ants are adept at conveying large food items away from competing ant species [35,42]. A scout that finds an item too large to move by itself can quickly assemble a transport group using a combination of acoustic and chemical recruitment signals [42,46]. The group then carries the load in a straight path over several meters back to the nest. Porters encircle the load, but they tend to favor its leading edge, where they walk backward and pull [35]. Although sometimes described as superefficient transporters, recent experiments show that this is not the case: because of their greater speed, lone porters carrying 0.3 g had roughly twice the prey delivery rate of 8-ant groups carrying 2.4 g (i.e., the same per capita weight) [35].

We experimentally tested the hypothesis that the reduced transport efficiency of groups is due to their diverse body alignments, either because some ants are required to work in inefficient postures or because the poorly aligned ants work against each other. We built an artificial load that forced all porters into the same orientation. We then measured transport speed, straightness, and group stability for different group sizes while keeping per capita load constant. We predicted that, if encirclement is a primary cause of inefficiency, we should not observe any decline in speed as group size increases. When this expectation was not met, we used simulations to evaluate the possibility that lower speed in groups might be explained by interactions among group members that vary in intrinsic transport speed.

## Methods

Cooperative transport was studied in three colonies of *N*. *cockerelli* living in South Mountain Park in Phoenix, AZ (33.335° N, 112.027° W). Experiments were carried out in June 2013 during the cooler early morning hours (05:30–07:30), when foragers were active outside the nest. Ants were induced to transport artificial loads (described below) across a Plexiglas platform (61 × 46 × 0.5 cm) resting directly on the ground 20 cm south of the main nest entrance. The platform was positioned horizontally (as determined by a spirit level) and covered with a single sheet of white paper.

**Fig 1:**
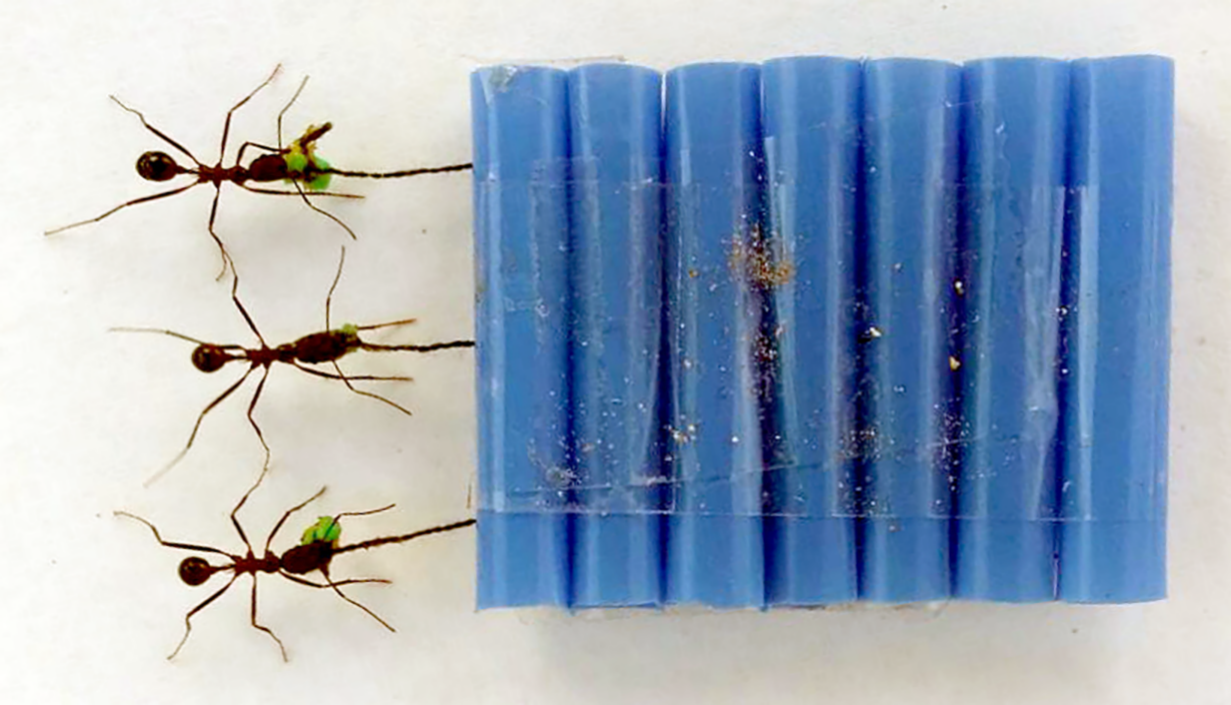
Artificial load used in experiments testing the effect of porter orientation on transport speed.

Before each experiment, foragers were attracted to the platform by placing a dried fig at its distal edge, 80 cm from the nest entrance. Once approximately ten foragers were present, the fig was replaced with an artificial load designed so that all porters had to pull it from the front, rather than encircling it. The load consisted of a 3 × 4 cm sled (constructed from segments of plastic drinking straws) that the ants could pull via 1-cm polyester threads attached to one edge (Fig 1). At the end of each thread was tied a 1 × 2 mm piece of ethylene vinyl acetate foam that offered an easy grasping point for a single ant. The foam was made attractive by lightly rubbing it against a dried fig. Because the sled itself was difficult for the ants to grasp, this design ensured that all porters would pull the load via the threads.

To systematically vary group size, we made loads with one, two, three, or four threads, distributed across the sled edge and separated from each other by 1 cm. Weights were added to each sled to achieve a constant individual load of 0.3 g per porter (i.e., load weights of 0.3, 0.6, 0.9, and 1.2 g for one, two, three, and four porters, respectively). For each group size, we performed 30 transport trials (10 for each colony). Each trial was recorded with a 720p high definition video camera (Canon G12) positioned above the center of the platform on a tripod (Manfrotto 055 Series). The field of view was shaded using an umbrella. A sample recording is available as supplementary material.

For each recording, we used ImageJ software [47] with the Mtrack plug-in [48] to record the location of the center of the sled at 10-s intervals. At each interval we also recorded the position of the head and gaster of every porter. We then focused our analysis on the longest recording segment during which group size remained stable for at least 10 s. This yielded 30, 24, 25, and 20 segments for 1-, 2-, 3-, and 4-ant teams, respectively. To avoid pseudoreplication, we tested only one segment from each trial. We estimated the path length of transport over this segment as the sum of straight-line distances between load positions measured every 10 s. Path length divided by the duration of the segment gave the average speed. We also measured displacement speed by taking the linear distance between the starting and ending positions of the load and dividing this value by the duration of the segment. We then compared the speed measures across group sizes using either analysis of variance (if assumptions of linear models were met) or a Kruskal-Wallis test.

Even using the sled design, porters varied somewhat in their orientation (see results below). We therefore tested whether the degree of porter alignment affected transport speed. At each sampling point, we represented the load's trajectory as a vector from its current position to its position at the next sampling point, 10 s later. We similarly represented the orientation of each porter as a unit vector parallel to the line connecting its head and gaster. We then projected the sum of these ant vectors onto the trajectory vector and divided the result by the number of porters. The resulting value served as an index of porter alignment, taking a maximum value of one if all porters were perfectly aligned with the load's trajectory. We then tested for a linear correlation between the alignment index and transport speed.

To describe the directness of transport we used the straightness index [49], calculated as the displacement of the load over the analyzed segment divided by the path length over the segment. This index has the advantage of simplicity and is expected to be a reliable estimator of the efficiency of oriented paths like those we analyze here [50]. Path straightness was compared among group sizes using a Kruskal-Wallis test.

To assess transport group stability we measured, for fully occupied sleds, the duration until the first ant left the group (or stopped pulling for solitary transport trials). We then fit Weibull functions to the distribution of group survival times for each group size. Weibull functions have two parameters: the scale parameter *λ* gives the mean rate at which the group breaks up (i.e., the first ant leaves), and the shape parameter *k* describes how this rate changes over time. If *k* = 1, the Weibull reduces to a simple exponential distribution, in which the rate of group breakup remains constant over time.

All statistical analyses and simulations were done using Matlab. A simulation script is given as supplemental material. For each measurement, we report average and standard deviation, except for variables that showed evidence of non-normality (Shapiro-Wilks test, α = 0.05), in which case we report the median and the 25^th^ and 75^th^ percentiles.

## Results

**Fig 2:**
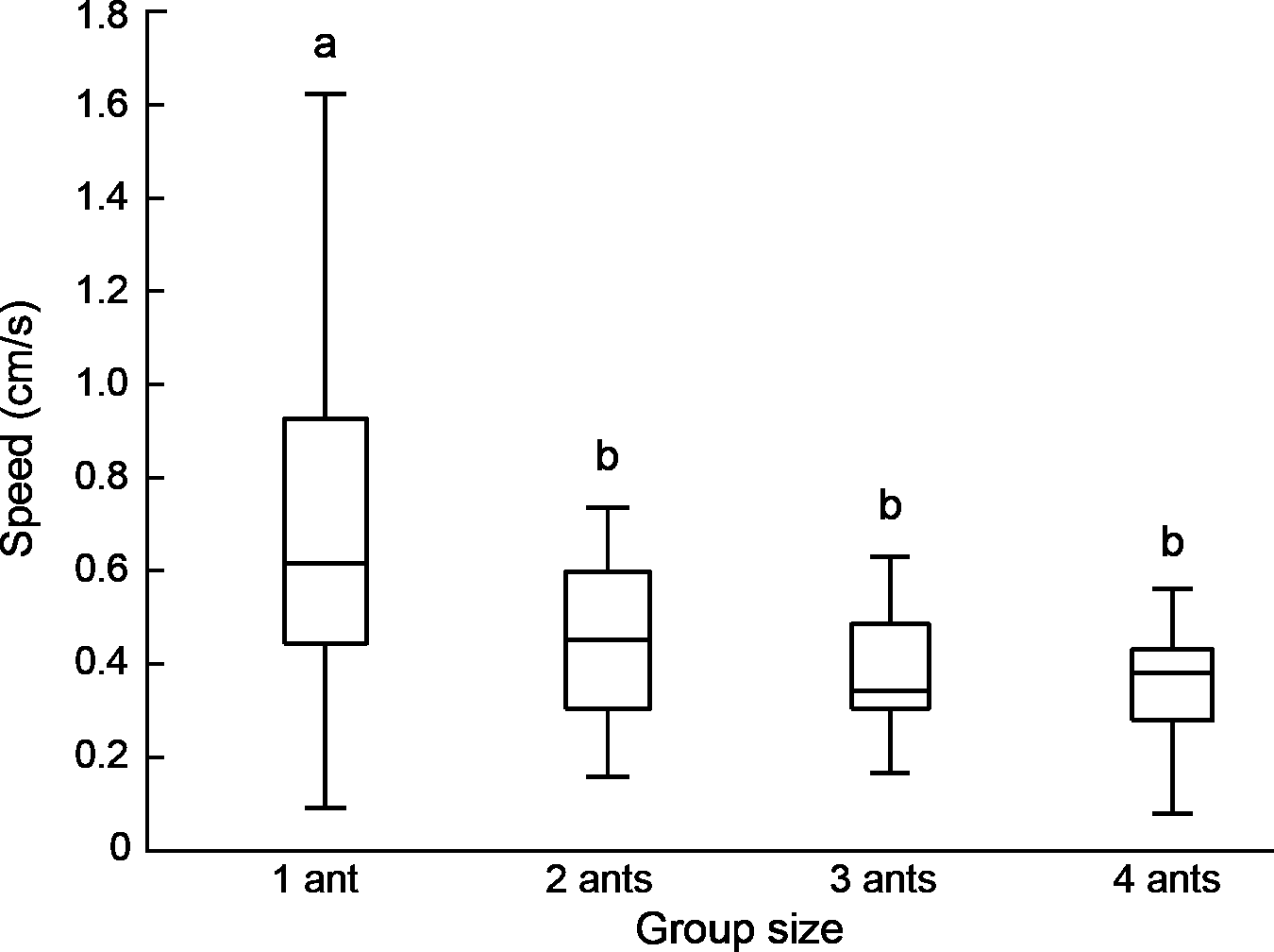
Speed of solitary and cooperative transporters with different group sizes. For all treatments, porters pulled the same per capita weight of 0.3 g. Mean speed differed significantly among treatments (ANOVA F_3, 95_ = 12.4, P<0.01). Treatments marked with different letters have significantly different mean speeds (Tukey tests, α = 0.05).

**Fig 3:**
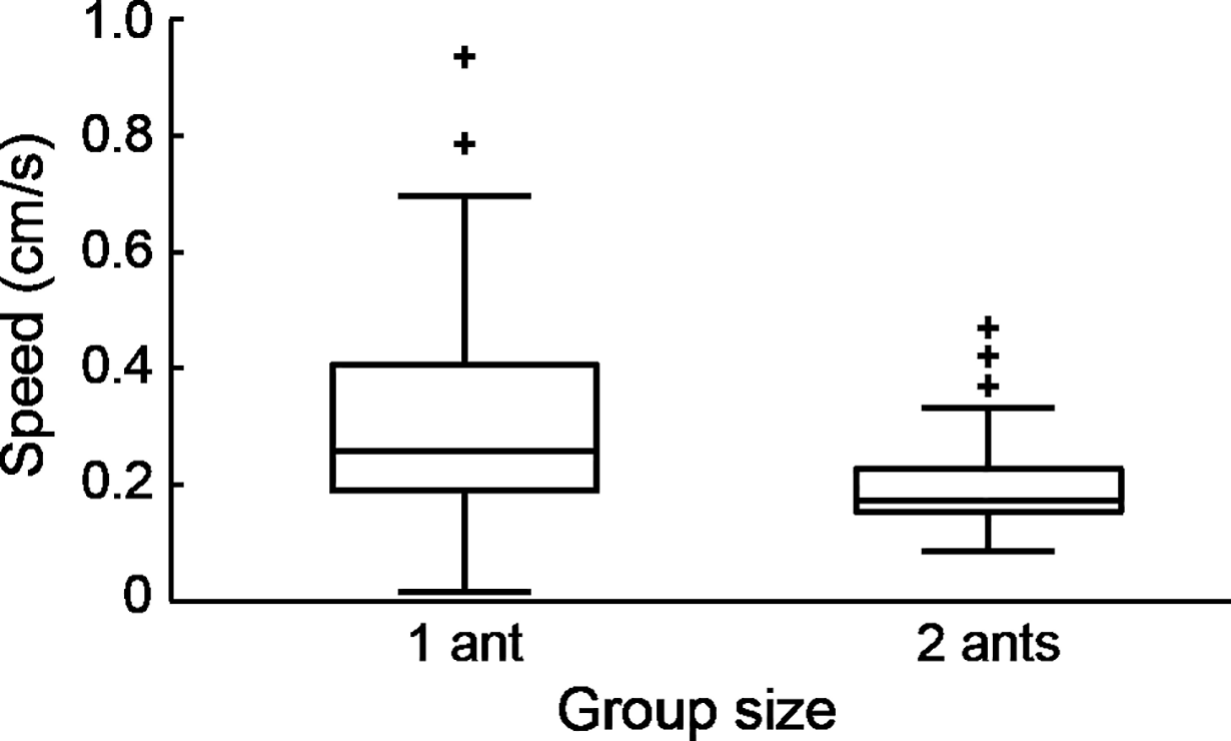
Speed of solitary and cooperative transporters pulling the same per capita weight of 0.6 g. Solitary ants (N = 27) pulled a 0.6 g sled intended for two ants, while pairs (N = 20) pulled a 1.2 g sled intended for four ants. Isolated individuals moved significantly faster (Mann-Whitney U = 223, P<0.01).

**Fig 4:**
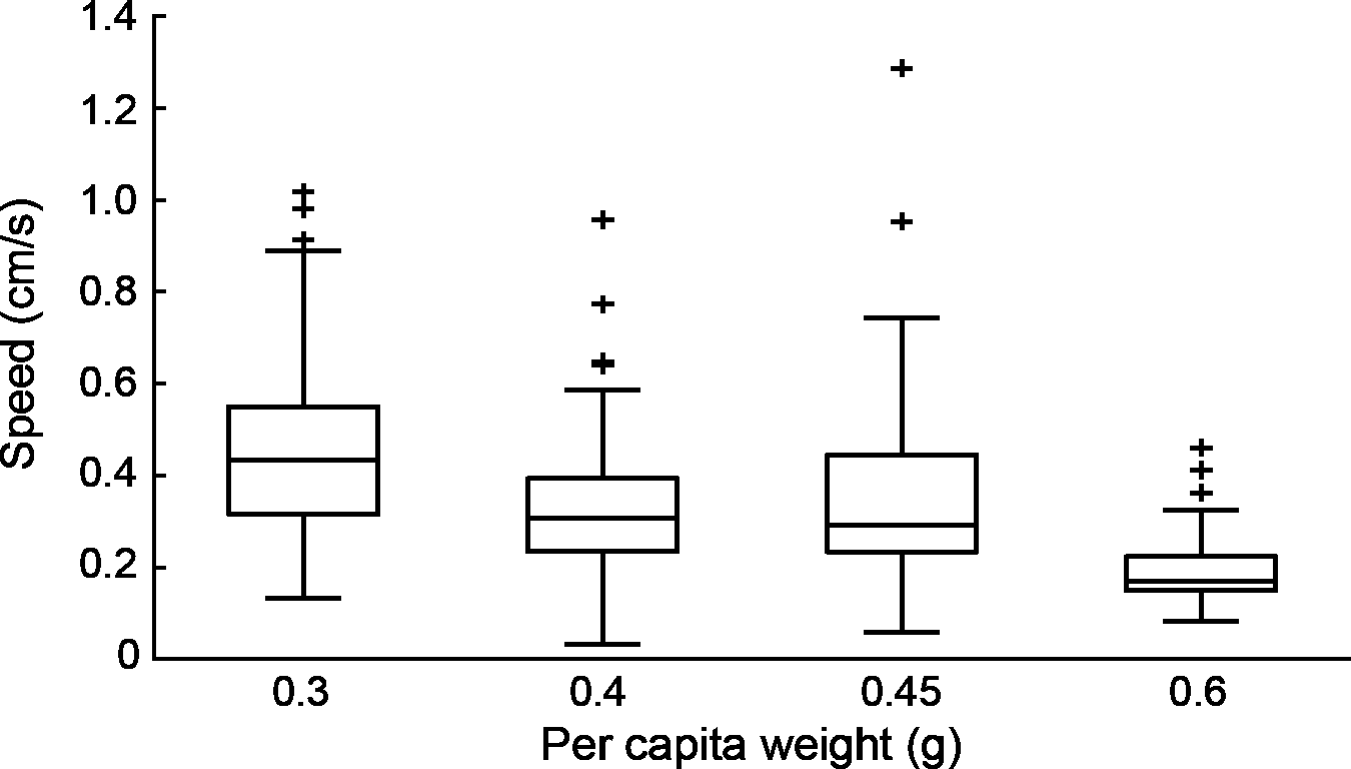
Effect of per capita weight pulled on the speed of cooperative transport. Data for each weight category were obtained as follows: 0.3 g: all fully-loaded 2-, 3-, and 4-ant sleds (N = 69); 0.4 g: 4-ant sleds pulled by only 3 ants (N = 24); 0.45 g: 3-ant sleds pulled by only 2 ants (N = 25); 0.6 g: 4-ant sleds pulled by only 2 ants (N = 20). For each trial we measured the speed over a 10 s interval during which the group remained intact.

Lone porters were faster than any group, with an average speed of 7.1 ± 3.7 mm/s, roughly twice that of groups (4.0 ± 1.5 mm/s for all groups combined). There was a small decline in average speed with increasing group size (4.6 ± 1.7, 3.8 ± 1.4, and 3.5 ± 1.3 mm/s for two-, three-, and four-ant groups, respectively), but this was not statistically significant (Fig 2). These results are for a per capita load of 0.3 g, but we found a similar pattern for a load of 0.6 g, observed when one or more threads were not occupied. That is, one ant pulling a two-thread sled weighing 0.6 g moved significantly faster than two ants pulling a four-thread sled weighing 1.2 g (Fig 3). Further analysis of partially occupied sleds allowed us to compare the speeds of groups pulling a range of different per capita loads. The results show that transport speed decreased as load increased (Fig 4; Spearman's r = -0.44, P<0.01).

In evaluating transport efficiency, we also considered path straightness, since slower transport speeds might be compensated by a straighter trajectory. Indeed, straightness varied with group size (Kruskal-Wallis test: H = 9.24, df = 3, P = 0.03), and lone porters had the most sinuous paths. However, differences among groups were very small, and transporters in all treatments took very straight paths, as indicated by average straightness indices close to one (1 ant: 0.97 [0.95–0.99], 2 ants: 0.99 [0.97–1.0], 3 ants: 0.99 [0.98–1.0] and 4 ants: 0.99 [0.98–1.0]). In fact, this advantage of path straightness for groups did not compensate for their lower speed. The displacement speed, which measures how fast workers reduced their distance from the nest entrance, was faster for solitary porters than for groups of three and four, and it did not vary among groups of different size (Kruskal-Wallis test: H = 20.42, df = 3, P = 0.0001; Nemenyi post-hoc test, α = 0.05). Median displacement speeds were 0.59 [0.44–0.96], 0.43 [0.30–0.58], 0.33 [0.30–0.48], and 0.37 [0.28–0.43] for one-, two-, three-, and four-ant treatments, respectively.

**Fig 5:**
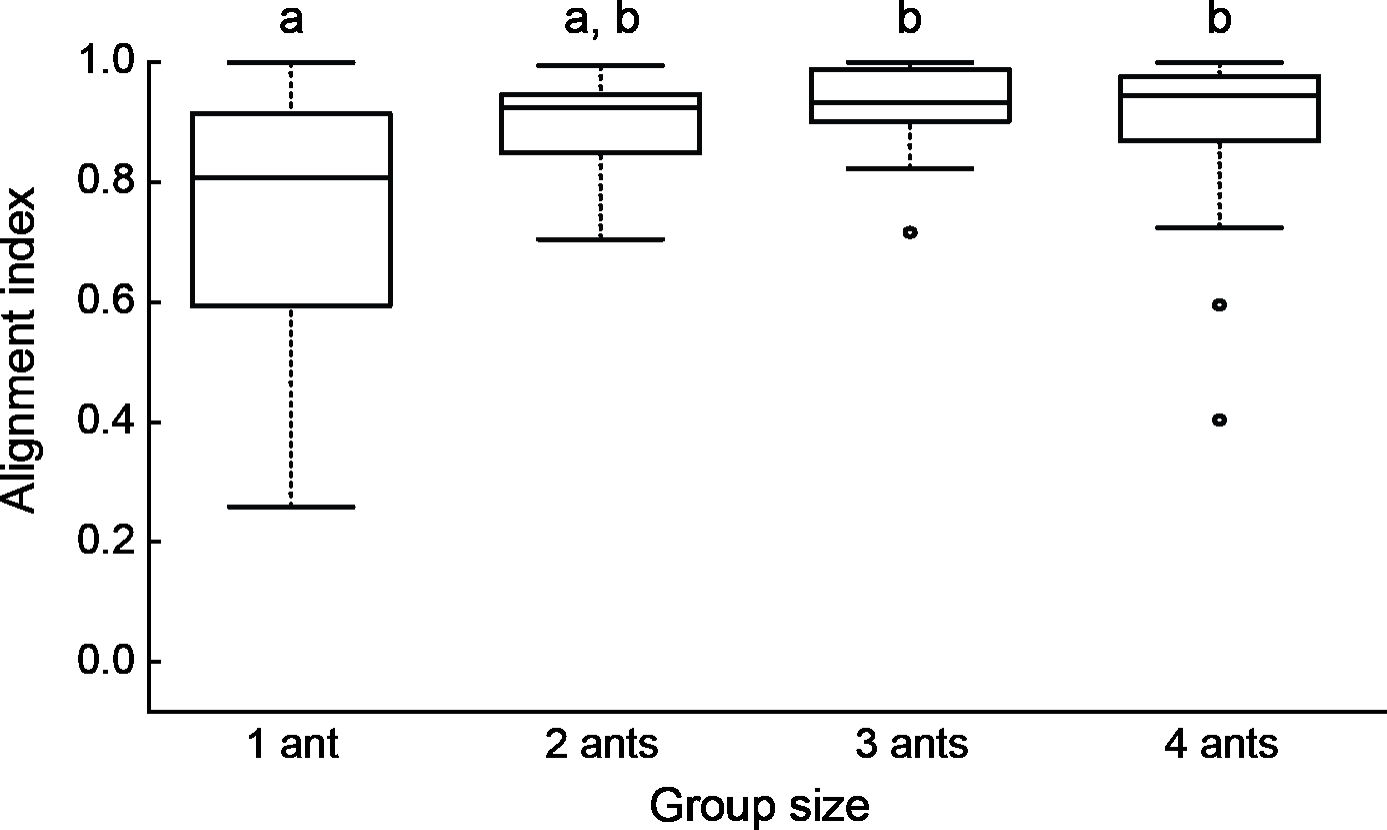
Relationship between group size and alignment index, a measure of how aligned porters are with each other and with the direction of transport (maximum value equals 1 for perfectly aligned groups). Alignment differed significantly among treatments (Kruskal-Wallis test: H = 16.6, df = 3, P<0.001). Treatments marked with different letters have significantly different alignment indices (Nemenyi tests, α = 0.05).

**Fig 6:**
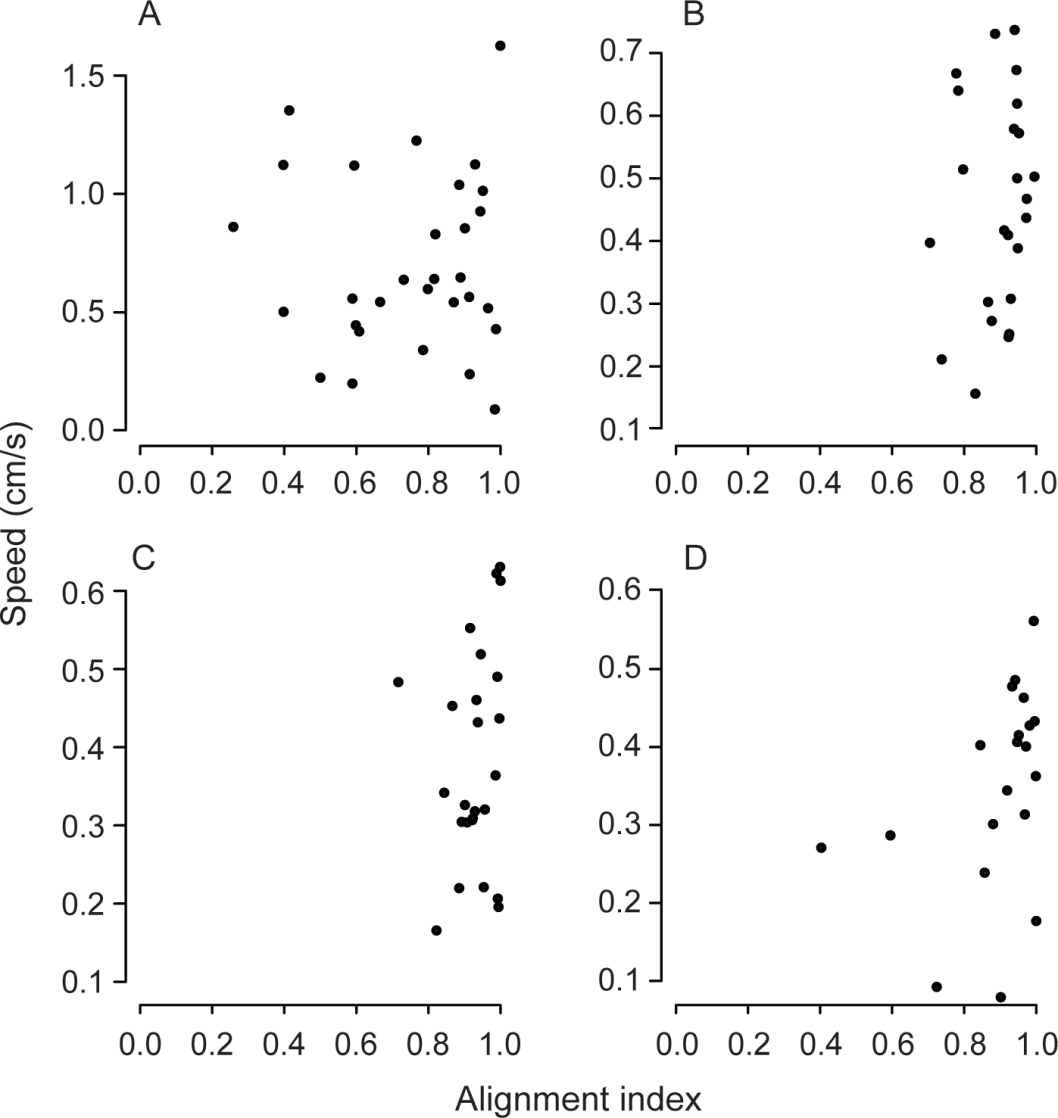
Relationship between transport speed and alignment index for solitary transporters (A) and teams of two (B), three (C), and four (D) ants. There was no significant correlation for any group size (Spearman's correlation: 1 ant: r = 0.03, P = 0.90; 2 ants: r = 0.23, P = 0.27; 3 ants: r = 0.29, P = 0.16; 4 ants: r = 0.43, P = 0.06).

To evaluate the influence of porter orientation on transport efficiency, we calculated an alignment index measuring how well the porters' body axes were aligned with each other and with the direction of movement. Porters generally showed high levels of alignment, with median indices of 0.8 or above for all group sizes, but there was marked variation among groups in the degree of alignment (Fig 5). We predicted that greater alignment should be associated with higher speeds, because of reduced cancellation of effort among group members. However, there was no significant correlation between the alignment index and the speed of transport for any group size (Fig 6). We also predicted greater alignment for groups than for solo porters, given the former’s greater speed. Again, this prediction was not met, as groups of size three and four were more aligned than solo ants. Other pairwise comparisons revealed no difference in alignment (Fig 5). These results contradict the hypothesis that groups are slower than individuals as a result of the ants exerting opposing forces.

The results of speed measurements on solitary porters revealed a high variance in speed, with values ranging from 1 to 16 mm/s (Fig 2). This suggested that group performance may be affected by interactions among group members of different ability. Specifically, we hypothesized that individual ants vary in walking speed and that groups are constrained to move at the speed of their slowest member. This might happen if a porter moving faster than its groupmates must slow down to avoid rotating the load, changing its trajectory, or breaking up the group. Such an effect would be consistent with the lower variance of speed in groups than in solitary porters (Fig 2).

To explore the viability of this hypothesis, we compared observed transport speeds with those of simulated groups composed of individuals with different intrinsic speeds. Each simulated ant's speed was drawn, with replacement, from the set of 30 observed lone porter speeds. The simulated group was then assigned the speed of its slowest member. For comparison, we also considered two other models for group interaction: 1) group speed equals the average speed of all members, and 2) group speed equals that of the fastest member. We created 10,000 simulated data sets and used them to calculate the expected average group speed under each model, as well as the probability of obtaining the average speed seen in the real data. The results show that observed average speeds were indistinguishable from those of simulated groups moving at the speed of the slowest member, but they were significantly different from the average speeds predicted by the other two models (Table 1). Observed variance in speed was significantly lower than predicted by any of the models, but it was closest to that of the slowest-member model.

**Table 1:** Comparison of observed transport speeds with those expected under three different models of interaction among porters. Observed speeds are given as the average ± standard deviation of group speed (cm/s) for three different group sizes. The three columns to the right give the corresponding values estimated from 10,000 simulated data sets. Each simulated set includes the same number of groups as the observed data (given by *n* in the column of observed speeds). P-values give the proportion of simulated average speeds that were more extreme than the observed average speed for each group size.

Group size	Observed speed	Speed of slowest member	Average speed of all members	Speed of fastest member
2	0.46±0.17 n = 24	0.50±0.26 P = 0.41	0.71±0.26 P<0.001	0.91±0.33 P<0.001
3	0.38±0.14 n = 25	0.41±0.21 P = 0.50	0.71±0.21 P<0.001	1.03±0.31 P<0.001
4	0.35±0.13 n = 20	0.36±0.18 P = 0.78	0.71±0.18 P<0.001	1.10±0.29 P<0.001

**Fig 7:**
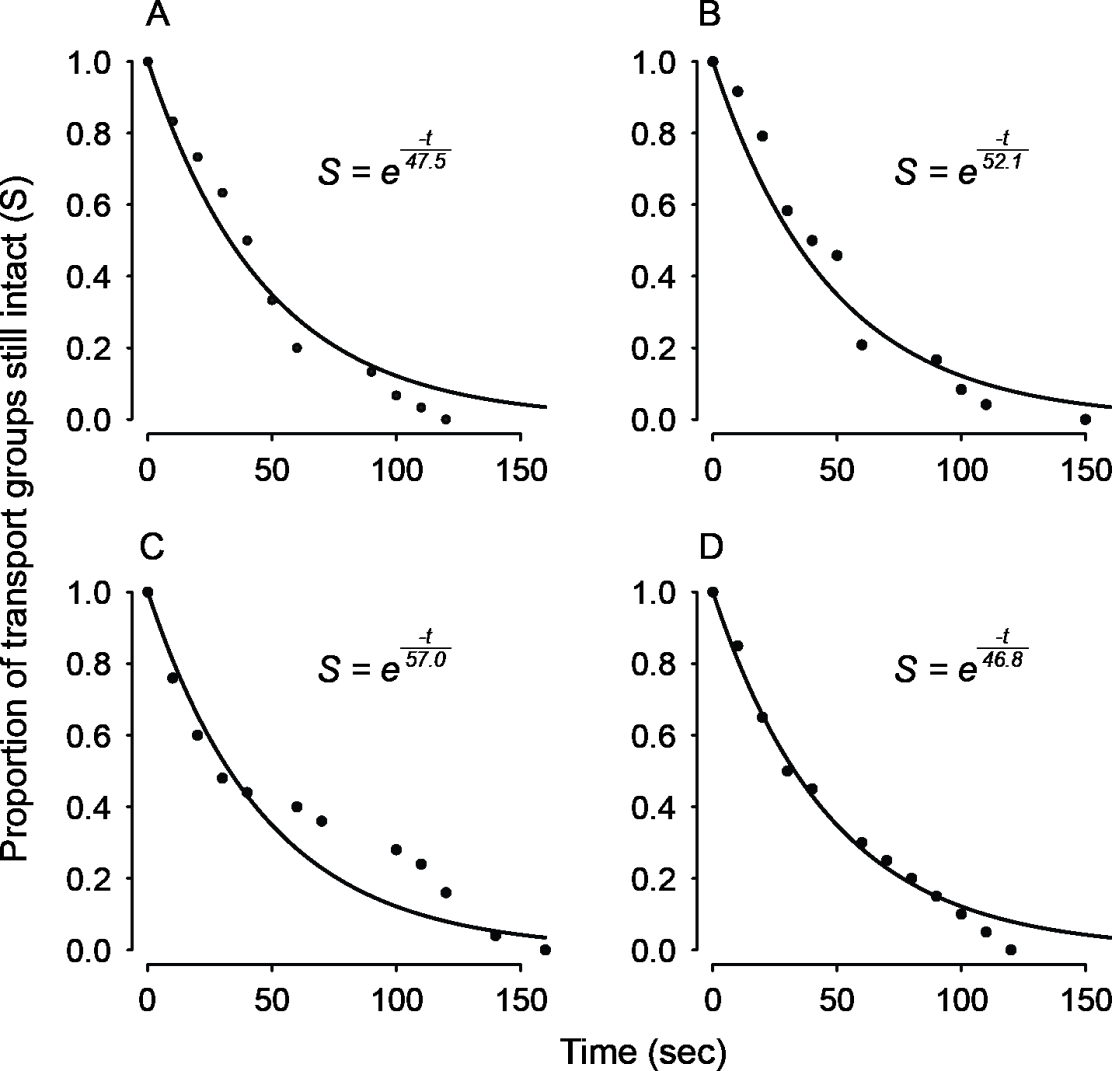
Distribution of transport persistence times for solitary transporters (A) and teams of two (B), three (C), and four (D) ants. Points show the proportion of transport teams still at their original size as a function of time since the start of observation. Their distributions are well approximated by exponential functions, implying that the probability of the first ant ceasing to transport is roughly constant over time. The denominator of the exponential term in each function gives the estimated mean time until the first departure, in seconds.

Transport efficiency is also expected to depend on group stability, because loss of a group member increases per capita weight and thus slows down transport. We therefore analyzed the distribution of transport group survival times: i.e., the duration until the first ant left the group. For each group size, the distribution of group survival times was adequately approximated by a simple exponential function, meaning that the probability of first departure from the group was roughly constant over the course of transport (Fig 7). Fig 7 also shows that this probability was quite similar across group sizes. This similarity at the group level implies differences at the individual level. If each ant had the same probability of leaving the group, regardless of group size, then we would expect group persistence to decline in proportion to group size. This is because the more ants are in a group, the more likely that one will leave at any given time. Instead, we saw nearly constant group persistence times across treatments, suggesting that an individual's probability of leaving the group decreases with group size.

## Discussion

Our results confirm earlier findings that solitary transport by *N*. *cockerelli* is faster and more direct than group transport when per capita loads are equal [35]. They further show that this difference persists even when all porters are made to adopt the same orientation, pulling the load and walking backwards. This result is not consistent with the hypothesis that groups are slower because of the range of orientations adopted by porters that encircle the load. If that hypothesis were true, we would have expected the speed difference to be substantially reduced or eliminated when all porters, both solitary and cooperative, were made to adopt the same orientation. In fact, groups remained much slower than individuals when this manipulation was carried out. In addition, we found no correlation between group speed and how well porters' body axes were aligned to each other and to the direction of travel.

We cannot conclude that transporter posture has no effect on transport speed—it remains possible that some orientations are more effective than others and that encircling the load therefore reduces speed. However, this effect, if present, is likely to be small. We infer this from a comparison of our results with those of the original study that found a slower speed for groups than individuals [35]. In that study, groups that encircled the load moved at an average speed of 2.5 mm/s vs. 5.3 mm/s for solitary porters, an absolute slowdown of 2.8 mm/s and a proportional one of 53%. In the present study, groups moved at an average speed of 4.0 mm/s vs. 7.1 mm/s for solitary porters, an absolute slowdown of 3.1 mm/s and a proportional one of 44%. Thus the drop in speed of groups vs. individuals is similar whether groups encircle the load or pull it in the same orientation as solitary porters. The higher overall speeds in the present study may reflect differences in the design of the loads used in the two experiments (e.g., the sleds in the current study might have lower friction with the substrate than the foam disks in the prior study).

An alternative explanation for slow cooperative transport was suggested by variation in the speed of lone porters. Many factors might affect an ant's speed, including its size, age, and experience. We can outline two general ways that such variation could in turn affect group speed. First, ants that are especially speedy may be less likely to join or remain in a transport group if, for example, slower individuals coordinate better or faster ones give up more easily. At present, we do not know if workers vary in their persistence or cooperative ability, or whether such variation is correlated with individual speed. This knowledge awaits further observations on individually-marked ants.

The second way that individual differences could reduce group speed is via interactions that restrain the speed of intrinsically faster ants. Our simulations support the feasibility of a scenario in which ants assemble into groups randomly with respect to their intrinsic speed, but then travel at the speed of the group’s slowest member. This model predicted the observed slowness of groups relative to lone ants, as well as a subtler reduction in speed as group size increased from two to four. The latter effect was not statistically significant, but this may reflect low power to detect a small effect. Group slowing might result from direct interactions among workers, with faster porters noting the actions of slower workers and adjusting their speed. However, it is more likely that interactions are mediated by the motion of the load itself. For example, a faster ant pulling at the edge of the load would generate a torque that rotates the load. If the ant detects this rotation, or simply feels increasing resistance as she pulls ahead of her teammates, she could respond by slowing down. Simple rules like this would lead to a stable state at which all faster ants had dropped their speed to that of the slowest one. While our simulations are consistent with this account, they do not offer a complete explanation of the phenomenon. In particular, the simulations overestimated the variance in group speed, implying an unrecognized factor that leads group speeds to be more similar than expected. Nonetheless, the simulations show the feasibility of a constraint on group speed imposed by the speed of the slowest ant. Rigorous testing of this idea will require experimental assembly of groups using ants with known intrinsic speeds.

It should be noted that slower groups are predicted only if per capita weight is held constant. This was the case in our experiments, because we gave proportionately heavier loads to larger teams. If team size is enlarged but load size remains the same, then the per capita weight declines and transport does not necessarily slow down. Indeed, we noted an increase in speed as with lower per capita weight (Fig 4), consistent with studies on other ants that show faster transport as team size increases for a given load [40,51,52].

The pattern we describe for ants has parallels in cooperative actions by other kinds of groups. The tendency for individuals to become less productive as their group size increases is known as the Ringelmann effect, after the author of classic work that showed this phenomenon in cattle and humans towing heavy loads [53–55]. Later work has provided other examples in humans [56,57], in other social animals [58], and even in robots [59]. Overall these studies conclude that cooperation broadens the array of achievable tasks and leads to faster task completion. However, group performance increases sub-linearly with group size, meaning that each additional member adds less to performance.

The Ringelmann effect can result when individuals work less hard in groups, relying instead on others to do the work (i.e., “social loafing”) [53,60,61]. However, the effect can arise even when individuals exert themselves fully, because of difficulties in coordinating larger groups. For example, experiments on ant-like robots cleaning a surface area showed that larger groups experienced increased interference and lower per capita work efficiency [59]. The speed conformity mechanism we propose above reflects a similar coordination challenge, since the group will break apart if workers do not settle on a common speed. However, we cannot rule out the possibility that ants change their effort depending on the presence of teammates. Future experiments can test this by comparing the forces exerted by porters when they work alone versus in a group.

Speed was not affected by group size, but larger groups showed greater cohesion than smaller ones, as shown by the decline in per capita rate of departure as group size increased. Individual persistence is crucial to the maintenance of group effort and the successful completion of cooperative transport. Once transport is underway, visible recruitment sharply decreases and few new ants join the transport team. Thus, if current porters left too readily, groups would soon disassemble, and transport would cease. In other group-living insects, aggregation and collective behavior are similarly facilitated by a positive influence of group size on an individual’s tendency to remain in the group [14,62]. Porters may respond directly to the presence of nestmates, perhaps mediated by short-range chemical and acoustic signals [42,46]. Alternatively, ants may instead perceive indirect cues related to team size. A potential cue is transport speed, but this seems unlikely, as the speeds of groups of different sizes could not be distinguished. Another possible cue is variation in speed over time. If individual ants exert force intermittently, then larger groups might experience less variation in the force exerted on the item and therefore its speed. Although all transports we observed seemed to move smoothly, further experiments would be needed to test this hypothesis.

In addition to its biological interest, cooperative transport in ants is a source of inspiration for swarm robotics [63,64]. Engineers are attracted to the ants’ ability to coordinate effectively without central control and in the face of obstacles and uneven terrain. The effects of group size on performance are likely to be of special importance for engineering applications. For ants, recruitment of extra porters is relatively cheap, but groups of robots may be more constrained in how large they can grow. Thus, it is important to understand how group size influences group efficiency, and what costs must be paid at the individual level to allow proper group coordination.

## Supporting information

S1 File Video of ants pulling artificial sleds. Retrieval teams pull the sled from the right side of the image toward their nest located to the left.
(MOV)

S2 File Script for simulation of group size effects on speed. Compressed file contains two files with Matlab script of simulation, one file with input data on individual speeds, and one file with output of simulation.
(ZIP)

S3 File Data on position of sleds for all replicates. (XLSX)

S4 File Data on position of sleds for each replicate in which pulling positions were fully occupied. (XLSX)

S5 File Data on position of ants for all replicates. (XLSX)

S6 File Data on position of ants for each replicate in which pulling positions were fully occupied. (XLSX)
